# First Report of Elevated Monocyte-Platelet Aggregates in Healthy Children

**DOI:** 10.1371/journal.pone.0067416

**Published:** 2013-06-24

**Authors:** Christina Yip, Vera Ignjatovic, Chantal Attard, Paul Monagle, Matthew D. Linden

**Affiliations:** 1 School of Medical Sciences, Royal Melbourne Institute of Technology University, Melbourne, Australia; 2 Murdoch Childrens Research Institute, Melbourne, Australia; 3 Department of Paediatrics, The University of Melbourne, Melbourne, Australia; 4 Department of Clinical Haematology, Royal Children’s Hospital, Melbourne, Australia; 5 Centre for Microscopy Characterisation and Analysis, The University of Western Australia, Perth, Australia; King’s College London School of Medicine, United Kingdom

## Abstract

Platelets are subcellular fragments which circulate in blood and have well established roles in thrombosis and haemostasis in adults. Upon activation, platelets undergo granule exocytosis and express P-Selectin on the cell membrane which binds a ligand on monocytes, leading to monocyte-platelet aggregation. Elevated circulating monocyte-platelet aggregates in adults are linked to atherothrombosis, but have not been investigated in children where thrombosis is less common. This study aimed to measure monocyte-platelet aggregate formation in children using whole blood flow cytometry. Monocyte-platelet aggregates as well as activation and granule exocytosis of platelets were measured in healthy adults (n = 15, median age 28 years) and healthy children (n = 28, median age 7 years). Monocyte-platelet aggregates in healthy children were elevated compared to healthy adults (37.8±4.4% vs 15.5±1.9% respectively, p<0.01). However, this was not accompanied by any difference in platelet activation (PAC-1 binding 6.8±1.5% vs 6.3±2.0% respectively, p = ns) or granule exocytosis (P-selectin expression 4.4±0.5% vs 3.1±0.5% respectively, p = ns). Despite comparable numbers of platelets bound per monocyte (GPIb MFI 117.3±13.7 vs 130.9±28.6 respectively, p = ns), surface P-selectin expression per platelet-bound monocyte was lower in children compared to adults. We therefore provide the first data of elevated monocyte-platelet aggregates in healthy children.

## Introduction

Platelets are small cell fragments of large importance in medicine. Their role in haemostasis and the late stage thrombotic complications of cardiovascular disease are well characterised [1]. However, platelets also play an important and central role in inflammation [2], with recently discovered antigen presenting capacity [3] and ability to influence the phenotype of other blood and vascular cells through cell-cell signalling [4], [5]. Monocyte-platelet aggregate (MPA) formation is a sensitive marker of platelet activation in adults and is an early marker of acute atherothrombotic events [6], [7]. The mechanism by which MPAs form in adults has been well characterized, where activated platelets which have undergone exocytosis express α-granule P-selectin (CD62P) on the cell surface. The platelet P-selectin then interacts with P-selectin glycoprotein ligand-1 (PSGL-1), which is constitutively expressed on the surface of circulating monocytes [8]. Following this initial tethering, the β2 integrin Mac-1 (CD11b/18), and to a lesser extent LFA-1 on the monocyte stabilise the adhesion [9]. However, these interactions do not develop if PSGL-1 is blocked, or CD62P is not expressed on the platelet [10], [11].

In addition to acting as a marker of platelet activation, the heterotypic cellular association between monocytes and platelets triggers an adhesive and pro-inflammatory monocyte phenotype [5], [12]. Although incompletely characterised, this is thought to arise both through outside-in signalling of the adhesion receptors interacting with the platelet surface, and through *in situ* delivery of pro-inflammatory platelet granule contents to the monocyte [5], [12]–[19]. Monocyte-platelet aggregates promote a pro-thrombotic milieu at the site of platelet activation, and are suggested to contribute to atherogenesis and progression of coronary artery disease (CAD) [5], [20], [21]. A potential role for sub-clinical platelet activation as a contributor to cardiovascular risk is emerging [12], [14].

Important age-related quantitative changes have been reported in haemostatic factors [22], [23] platelet count [24], [25] and reactivity [26], [27] among children, including decreasing soluble P-selectin in serum with age [28]. These age-related changes in haemostasis have important implications in the clinical management of children [23]. Measurement of monocyte-platelet aggregates is used as a surrogate marker of early platelet activation in paediatric research for many thromboinflammatory diseases, including acute myocardial infarction, cystic fibrosis and thrombocytopenia [14], [29], [30]. However, the formation of MPAs in children and their circulating levels has not been systematically investigated in healthy children. We therefore sought to measure formation of MPAs in healthy children, and compare them with adults.

## Results

**Figure 1:**
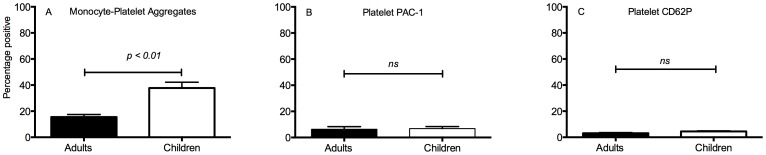
Elevated circulating monocyte-platelet aggregates (MPAs) in children with no increase in platelet activation or exocytosis. Monocytes were identified by characteristic forward and side laser scatter and differential expression of CD14, while MPAs were determined by co-expression of platelet-specific GPIX (CD42a) on monocyte events and gates were determined by appropriate isotypic control. The percentage of monocyte-platelet aggregates as a function of overall monocytes was recorded (Panel A) Platelets were identified by characteristic forward and side laser scatter and expression of platelet-specific GPIbα, with a threshold discriminator on CD42b-PC5. The percentage of platelets with PAC-1 FITC fluorescence above the eptifibatide-blocked control (panel B) or P-selectin expression above isotype control was recorded (panel C). Data shown are mean +/− SEM (adults n = 15, children n = 28).

Circulating MPAs were increased in children compared to adults as shown in Figure 1A (37.8±4.4% vs. 15.5±1.9% respectively, p<0.01). However, when circulating platelets were examined for surface markers of activation with no addition of agonists *ex vivo*, there was no corresponding increase in activation of the GPIIb/IIIa receptor as measured by PAC-1 binding (6.8±1.5% vs. 6.3±2.0% respectively, p = ns) (Figure 1B); or platelet granule exocytosis, as measured by P-selectin expression (4.4±0.5% vs. 3.1±0.5% respectively, p = ns) (Figure 1C).

**Figure 2:**
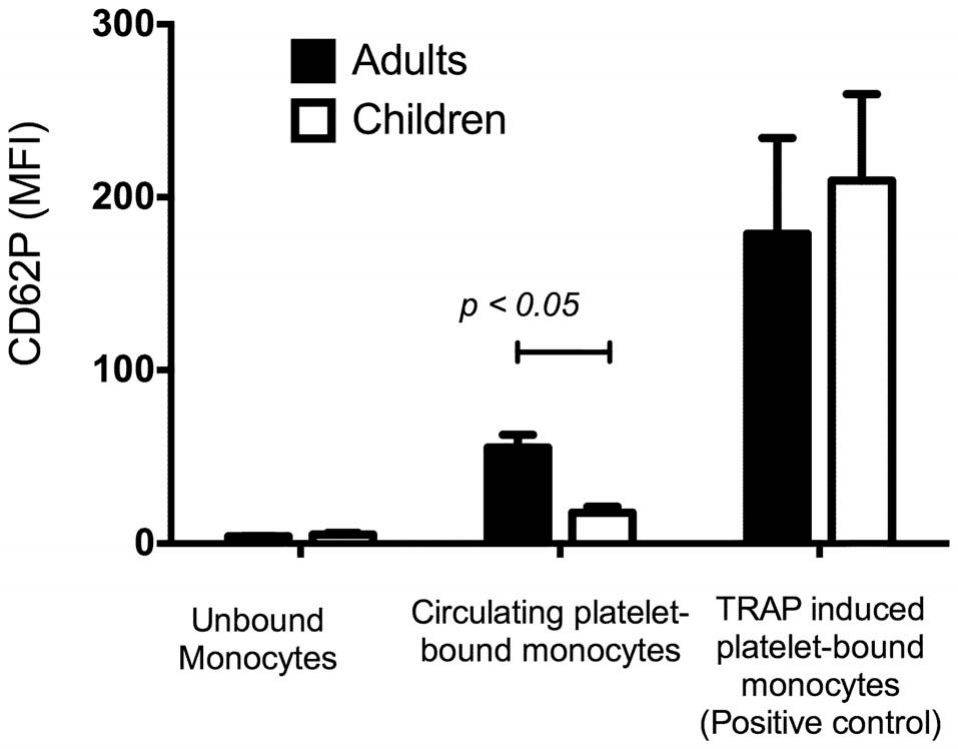
Expression of P-selectin (CD62P) mean fluorescence intensity (MFI) of monocytes with and without platelets bound, with and without the chemical platelet agonist TRAP-6 (positive control) in children and adults. Circulating MPAs in children had lower P-selectin expression than adults, but could be induced to express P-selectin by chemical stimulation. Data shown are mean MFI +/− SEM (adults n = 4 and children n = 4).

In order to determine whether circulating monocyte-platelet aggregates in children formed as a result of the P-Selectin/PSGL-1 adhesion mechanism known to be responsible for this process in adults, the relative MFI of P-selectin on platelet-bound and –unbound monocyte events was examined. P-selectin expression of platelets bound to circulating monocytes in blood from children have significantly lower P-selectin expression compared to platelets bound to monocytes in circulating blood from adults (17.8±3.5 vs. 58.5±15.8 MFI respectively, p<0.05) (Figure 2). However, *ex vivo* chemical stimulation of whole blood to with 50 µM TRAP-6, a specific agonist of the platelet protease activated receptor, resulted in equal expression of P-selectin on monocyte-platelet aggregates between adults and children.

In order to compare the relative number of platelets bound per monocyte without *ex vivo* chemical stimulation, the expression of platelet-specific GPIX (CD42a) was compared. Mean fluorescent intensity of CD42a from children (117.3±13.7 MFI) was not different to adults (130.9±28.6 MFI, p = ns), indicating similar number of platelets bound per monocyte.

## Discussion

We provide the first evidence of elevated MPAs without ex vivo chemical stimulation in children. Platelets bound to monocytes in children did not show the elevation of P-selectin expression associated with MPA formation in adults. These results suggest that circulating MPAs in children are not a result of increased platelet activation and granule exocytosis, and that a P-selectin independent mechanism of MPA formation may be more important in MPA formation in children. These findings are very different to what has been described in adults. This is the first report of such age-specific differences in monocytes platelet interaction and it is consistent with our previous findings of age-dependent changes in other haemostatic and platelet parameters.

The lower P-selectin expression on circulating MPAs in children was not associated with a corresponding decrease in CD42a expression, indicating similar numbers of platelets bound per monocyte in MPAs from children and adults. Further testing by stimulation with a supra-maximal concentration of the chemical platelet agonist TRAP-6 demonstrated that platelets bound to monocytes from both children and adult blood could be induced to a similar maximal expression of P-selectin. Therefore, while the majority of circulating MPAs form in the absence of platelet activation in children, there is the capacity for both platelet activation-dependent and -independent formation of monocyte-platelet aggregates. This also confirms that the binding of anti-CD62P monoclonal antibodies to platelets that are already bound through P-Selectin/PSGL-1 mechanisms is not impaired in our assay.

Age related differences in relative abundance of monocytes and platelets [24], [25], [31] might potentially contribute to the observed differences in the level of MPAs in blood from children. In order to test this hypothesis, we collected and analysed full blood counts and MPAs on a subset of samples from 5 adults and 6 children (data not shown). No correlation (Spearman’s rank test) of MPAs with either absolute platelet count, monocyte count or the ratio of platelet to monocyte count was observed.

Pre-analytical variables, especially with relation to blood collection, are known to affect parameters of platelet function, including MPAs. We performed preliminary experiments using different sample collection methods using direct venipuncture compared with blood drawn through indwelling catheter. This confirmed the need for blood collection procedures to be consistent, and so blood from healthy adult volunteers was drawn through an indwelling catheter to match routine paediatrics collection in this setting. Bloods were collected using the same peripheral cannula type and size into the same type of anticoagulant tubes. With this stringent standardization, difference in MPAs formation we reported here is unlikely attributed by the pre-analytical variables.

While not routinely used as a diagnostic assay in clinical medicine, whole blood flow cytometric measurement of monocyte-platelet aggregates is considered an early and sensitive marker of *in vivo* platelet activation and atherothrombosis in adults [6], [9], and proposed as a potential diagnostic tool in the assessment of acute coronary syndromes [9]. Monocyte-platelet aggregates have also been used as a sensitive marker of platelet activation in the context of thromboinflammatory disease state of relevance to paediatric populations, such as cystic fibrosis [29]. However, in the current study circulating monocyte-platelet aggregates observed in healthy children were not associated with platelet activation, and the adherent platelets had not undergone exocytosis. Therefore care must be taken in the use of MPAs as a marker of platelet activation in children.

While MPAs in adults are thought to contribute to atherogenesis through promotion of an inflammatory phenotype and by localizing monocytes to the endothelium, circulating MPAs in children are unlikely to play a role in this process in the absence of P-selectin and platelet activation. Further research should be directed at understanding the different mechanisms and phyisiological consequences of monocytes-platelets interaction in children. The potential role for this physiological adhesion between unactivated platelets and monocytes in the protection of children from a number of clinically significant platelet mediated diseases such as heparin induced thrombocytopenia [32] deserves further exploration.

It is well established in adult blood that following initial tethering to activated platelets by P-selectin/PSGL-1 interaction, firm adhesion via bridging fibrinogen bound to the activated GPIIb-IIIa integrin [33] and via direct interaction with GPIbα [34]. Our finding of elevated monocyte-platelet tethering in children without elevated P-Selectin expression therefore warrants further investigation into the role and relative importance of other adhesion molecules, such as Mac-1, LFA-1 and ICAM-2, which may have a role in the in the observed formation of circulating monocyte-platelet aggregates in the absence of P-selectin.

Following granule exocytosis and exposure of P-selectin on activated platelets, P-selectin is rapidly cleaved while activated platelets continue to circulate and function [35]. It is therefore possible that the elevated circulating MPAs in children represent platelets that are initially bound to monocytes *in vivo* through P-selectin expression and that subsequently, but prior to blood collection, P-selectin is cleaved off with the platelet remaining tethered to the monocyte through other adhesion molecules.

In summary, this is the first observation of increased monocyte-platelet aggregation occurs in healthy children compared to healthy adults. This interaction occurs without the increased expression of P-selectin seen with MPA formation in adults, suggesting a different physiological monocyte-platelet interaction in this young population. This interaction could contribute to the thromboprotective mechanism observed in children. Further investigation of the phenotypic consequences of platelet adhesion to monocytes in the absence of P-selectin is warranted.

## Materials and Methods

### Participants and Blood Collection

Institutional human research ethics approval (RMIT University Human Research Ethics Committee reference 55/11 and Royal Children’s Hospital Melbourne Human Research Ethics Committee reference 20031) was obtained for the research and the procedure of gaining consent. For adults, written informed consent was obtained, while for children both verbal assent from the child and written consent from a parent were obtained. Blood from healthy volunteer adults (n = 15, age 20–43 years old, median age 28 years old) and healthy children of either gender (n = 28, age 1–14 years old, median age 7 years old) scheduled for minor day surgery (e.g. trigger thumb release) was collected. Subjects did not receive any antiplatelet medication and had no family history of haematological disorders. Whole blood was collected from peripheral cannula into S-Monovette tubes (Sarstedt, Australia), containing 1 volume of citrate per 9 volumes of blood according to protocols previously described [22]. In order to minimise pre-analytical variables, blood collection procedures and blood handling for adults and children were identical.

### Assays

#### Flow cytometer setup and calibration

**Figure 3:**
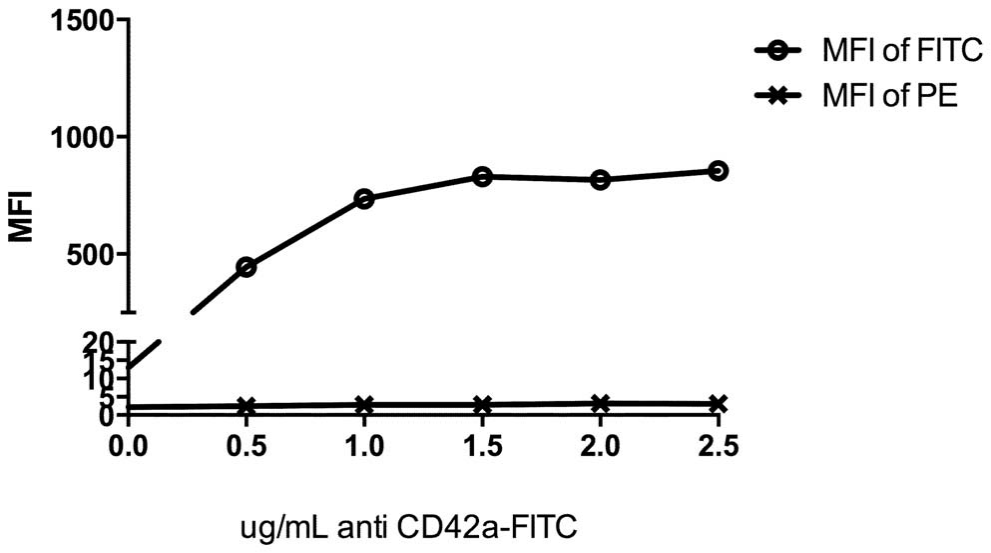
Compensation of spectral overlap for FITC and PE. Compensation matrices were determined with single colour controls of high and low expressing cells and accurate compensation confirmed by antibody titration.

All analyses were performed on a FACS Calibur flow cytometer (Becton Dickinson, Australia). Fluorophores were excited by a 30 mW 488 nm argon sapphire laser. Fluorescein isothiocyanate (FITC) emission was collected in the wavelength range 515–545 nm (530/30 band pass), phycoerythrin (PE) emission was collected in the wavelength range 564–606 nm (585/42 band pass) and tandem phycoerythrin-cy5 (PE-Cy5) emission was collected in the wavelength >670 nm (670 long pass). Voltages to photomultiplier tubes (PMTs) collecting each wavelength range were established to ensure emission peaks for unstained and positively stained cells fell within the linear range of the instrument (i.e. between first and third decades of MFI). Voltages were individually calibrated for platelet and monocyte-platelet parameters against hard-dyed broad spectrum 3.0–3.4 µm 8 peak calibration particles of known concentration of molecules of equivalent soluble fluorochrome (MESF) (Spherotech, USA) as previously described [36]. Briefly, for each day in which samples were analysed, 8 peak rainbow beads were first analysed and PMT voltages adjusted such that each peak of fluorochrome labelled beads of known MESF appear in the appropriate channel for each bandwidth range. Compensation for spectral overlap between all fluorophores was determined using platelets and monocytes with high and low expression individually single stained with antibody. A mixed population of high and low expressing cells was created by partial activation of platelets using 2 µM TRAP-6. Effective compensation was verified by antibody titration on high expressing cells (Figure 3).

#### Measurement of platelet activation and exocytosis

Platelet activation was measured by whole blood flow cytometric determination of PAC-1 binding, a monoclonal antibody which recognizes integrin αIIbβ3 in its high affinity activated conformation, indicating activation dependent inside-out signalling has occurred. Granule exocytosis was measured by expression of P-selectin, an α-granule component not normally expressed on the surface of resting platelets but expressed following fusion of granule and cell membranes. Briefly, within 30 min of collection whole blood was diluted 1∶5 with HEPES saline (10 mM HEPES, 0.15 M NaCl, pH 7.3–7.4) and incubated with an antibody cocktail containing 1.0 µg.mL^−1^ PC5 conjugated anti-CD42b monoclonal antibody (clone HIP1, Becton Dickinson Pharmingen, USA), 6.25 µg.mL^−1^ FITC conjugated PAC-1 (Becton Dickinson, Australia) with or without blockade by 5 µg.mL^−1^ eptifibatide (Schering-Plough, Australia), and PE conjugated anti-CD62P monoclonal antibody (clone AK4, Becton Dickinson, Australia) or 1 µg.mL^−1^ PE conjugated mouse IgG1κ isotypic control (clone MOPC-21, Becton Dickinson, Australia). After 15 min of incubation the reaction was stopped with addition of 800 µL of 1% formaldehyde (Sigma Aldrich, USA) in HEPES-Saline. Platelets activated with 50 µM of thrombin receptor activating peptide, TRAP-6 (Sigma Aldrich, Australia) were analysed with each sample as a positive control. Platelets were identified by characteristic forward and side laser scatter and expression of platelet-specific GPIbα (CD42b). The percentage of platelets with fluorescence above the eptifibatide-blocked or isotype controls was recorded.

#### Measurement of Monocyte-Platelet Aggregate (MPA) formation

Circulating MPAs were measured by whole blood flow cytometry with previously described methods [1]. Briefly, within 20 minutes of collection whole blood was incubated with an antibody cocktail containing 1.0 µg.mL^−1^ FITC conjugated anti-CD42a monoclonal antibody (clone ALMA.16, Becton Dickinson, Australia) or 1.67 µg.mL^−1^ FITC conjugated mouse IgG1κ isotypic control and 0.5 µg.mL^−1^ PC5 conjugated anti-CD14 monocloncal antibody (clone RMO52, Beckman Coulter Immunotech, Australia) with or without TRAP-6. After 15 minutes of incubation, the reaction mixture was stopped with 800 µL of FACS Lysing solution (Becton Dickinson, Australia) and stored at 2–8°C in the dark until analysis.

Monocytes were identified by characteristic forward and side laser scatter and differential expression of CD14, while MPAs were determined by co-expression of platelet-specific GPIX (CD42a) on monocyte events. Gates were determined by appropriate isotypic control. In a subset of samples, P-selectin expression of monocyte-bound platelets was quantified by addition of CD62P-PE (clone AK-4) to the antibody cocktail, with sequential gating and fluorochrome compensation as previously described [5].

### Statistical Analysis

Data shown are mean +/− SEM. Results for children and adults were compared using the non-parametric Wilcoxon rank sum test.

## References

[1] Linden MD, Frelinger AL, 3rd, Barnard MR, Przyklenk K, Furman MI, et al (2004) Application of flow cytometry to platelet disorders. Semin Thromb Hemost 30: 501–511. 15497093 10.1055/s-2004-835671

[2] Smith TL, Weyrich AS (2011) Platelets as central mediators of systemic inflammatory responses. Thromb Res 127: 391–394. 21074247 10.1016/j.thromres.2010.10.013 PMC3081926

[3] Chapman LM, Aggrey AA, Field DJ, Srivastava K, Ture S, et al (2012) Platelets present antigen in the context of MHC class I. J Immunol. 189: 916–923. 10.4049/jimmunol.1200580 PMC3392496 22706078

[4] Linden MD, Furman MI (2005) Monocyte-platelet aggregates in patients with ischemic heart disease In: Morrow DA, Cannon C, editors. Contemporary Cardiology: Cardiac Biomarkers in the Management of Cardiovascular Disease. Totowa, New Jersey: Humana Press. 487–493.

[5] Barnard MR, Linden MD, Frelinger AL, 3rd, Li Y, Fox ML, et al (2005) Effects of platelet binding on whole blood flow cytometry assays of monocyte and neutrophil procoagulant activity. J Thromb Haemost 3: 2563–2570. 16241954 10.1111/j.1538-7836.2005.01603.x

[6] Michelson AD, Barnard MR, Krueger LA, Valeri CR, Furman MI (2001) Circulating monocyte-platelet aggregates are a more sensitive marker of in vivo platelet activation than platelet surface P-selectin: studies in baboons, human coronary intervention, and human acute myocardial infarction. Circulation 104: 1533–1537. 11571248 10.1161/hc3801.095588

[7] Linden MD, Furman MI, Frelinger AL, 3rd, Fox ML, Barnard MR, et al (2007) Indices of platelet activation and the stability of coronary artery disease. J Thromb Haemost 5: 761–765. 17371489 10.1111/j.1538-7836.2007.02462.x

[8] Furie B, Furie BC (1995) The molecular basis of platelet and endothelial cell interaction with neutrophils and monocytes: role of P-selectin and the P-selectin ligand, PSGL-1. Thromb Haemost 74: 224–227. 8578462

[9] Linden MD, Furman MI (2006) Monocyte-Platelet Aggregates in Patients With Ischemic Heart Disease. In: Morrow DA, editor. Cardiovascular Biomarkers: Pathophysiology and Disease Management. Totowa, NJ: Humana Press. 487–493.

[10] Larsen E, Celi A, Gilbert GE, Furie BC, Erban JK, et al (1989) PADGEM protein: a receptor that mediates the interaction of activated platelets with neutrophils and monocytes. Cell 59: 305–312. 2478294 10.1016/0092-8674(89)90292-4

[11] Hamburger SA, McEver RP (1990) GMP-140 mediates adhesion of stimulated platelets to neutrophils. Blood 75: 550–554. 1688717

[12] Linden MD, Jackson DE (2010) Platelets: pleiotropic roles in atherogenesis and atherothrombosis. Intl J Biochem Cell Biol 42: 1762–1766. 10.1016/j.biocel.2010.07.012 20673808

[13] Hidari KI, Weyrich AS, Zimmerman GA, McEver RP (1997) Engagement of P-selectin glycoprotein ligand-1 enhances tyrosine phosphorylation and activates mitogen-activated protein kinases in human neutrophils. J Biol Chem 272: 28750–28756. 9353345 10.1074/jbc.272.45.28750

[14] Lanza GA, Scalone G, Barone L, Infusino F, Coviello I, et al (2011) Platelet reactivity and endothelial function in children of patients with early acute myocardial infarction. Eur Heart J 32: 2042–2049. 21565849 10.1093/eurheartj/ehr109

[15] Blanks JE, Moll T, Eytner R, Vestweber D (1998) Stimulation of P-selectin glycoprotein ligand-1 on mouse neutrophils activates beta 2-integrin mediated cell attachment to ICAM-1. Eur J Immunol 28: 433–443. 9521050 10.1002/(SICI)1521-4141(199802)28:02<433::AID-IMMU433>3.0.CO;2-U

[16] Evangelista V, Manarini S, Sideri R, Rotondo S, Martelli N, et al (1999) Platelet/polymorphonuclear leukocyte interaction: P-selectin triggers protein-tyrosine phosphorylation-dependent CD11b/CD18 adhesion: role of PSGL-1 as a signaling molecule. Blood 93: 876–885. 9920836

[17] Lindmark E, Tenno T, Siegbahn A (2000) Role of platelet P-selectin and CD40 ligand in the induction of monocytic tissue factor expression. Arterioscler Thromb Vasc Biol 20: 2322–2328. 11031222 10.1161/01.atv.20.10.2322

[18] Christersson C, Johnell M, Siegbahn A (2008) Tissue factor and IL8 production by P-selectin-dependent platelet-monocyte aggregates in whole blood involves phosphorylation of Lyn and is inhibited by IL10. J Thromb Haemost 6: 986–994. 18363812 10.1111/j.1538-7836.2008.02956.x

[19] Celi A, Pellegrini G, Lorenzet R, De Blasi A, Ready N, et al (1994) P-selectin induces the expression of tissue factor on monocytes. Proc Natl Acad Sci U S A 91: 8767–8771. 7522321 10.1073/pnas.91.19.8767 PMC44687

[20] Totani L, Evangelista V (2010) Platelet-leukocyte interactions in cardiovascular disease and beyond. Arterioscler Thromb Vasc Biol 30: 2357–2361. 21071701 10.1161/ATVBAHA.110.207480 PMC3076621

[21] Gawaz M (2006) Platelets in the onset of atherosclerosis. Blood Cells, Molec Dis 36: 206–210. 16476558 10.1016/j.bcmd.2005.12.022

[22] Ignjatovic V, Lai C, Summerhayes R, Mathesius U, Tawfilis S, et al (2011) Age-related differences in plasma proteins: how plasma proteins change from neonates to adults. PLoS ONE 6: e17213. 21365000 10.1371/journal.pone.0017213 PMC3041803

[23] Monagle P, Barnes C, Ignjatovic V, Furmedge J, Newall F, et al (2006) Developmental haemostasis. Impact for clinical haemostasis laboratories. Thromb Haemost 95: 362–372. 16493500 10.1160/TH05-01-0047

[24] Biino G, Santimone I, Minelli C, Sorice R, Frongia B, et al (2013) Age- and sex-related variations in platelet count in Italy: a proposal of reference ranges based on 40987 subjects’ data. PLoS ONE 8: e54289. 23382888 10.1371/journal.pone.0054289 PMC3561305

[25] Segal JB, Moliterno AR (2006) Platelet counts differ by sex, ethnicity, and age in the United States. Ann Epidemiol 16: 123–130. 16246584 10.1016/j.annepidem.2005.06.052

[26] Ignjatovic V, Than J, Summerhayes R, Newall F, Horton S, et al (2012) The quantitative and qualitative responses of platelets in pediatric patients undergoing cardiopulmonary bypass surgery. Pediatr Cardiol 33: 55–59. 21809131 10.1007/s00246-011-0079-5

[27] Hezard N, Potron G, Schlegel N, Amory C, Leroux B, et al (2003) Unexpected persistence of platelet hyporeactivity beyond the neonatal period: a flow cytometric study in neonates, infants and older children. Thromb Haemost 90: 116–123. 12876634

[28] Ponthieux A, Herbeth B, Droesch S, Lambert D, Visvikis S (2003) Age- and sex-related reference values for serum adhesion molecule concentrations in healthy individuals: intercellular adhesion molecule-1 and E-, P-, and L-selectin. Clin Chem 49: 1544–1546. 12928244 10.1373/49.9.1544

[29] O’Sullivan BP, Linden MD, Frelinger AL, 3rd, Barnard MR, Spencer-Manzon M, et al (2005) Platelet activation in cystic fibrosis. Blood 105: 4635–4641. 15705796 10.1182/blood-2004-06-2098

[30] Haselboeck J, Pabinger I, Ay C, Koder S, Panzer S (2012) Platelet activation and function during eltrombopag treatment in immune thrombocytopenia. Ann Hematol 91: 109–113. 21553010 10.1007/s00277-011-1249-5

[31] Lugada ES, Mermin J, Kaharuza F, Ulvestad E, Were W, et al (2004) Population-based hematologic and immunologic reference values for a healthy Ugandan population. Clin Diagn Lab Immunol 11: 29–34. 14715541 10.1128/CDLI.11.1.29-34.2004 PMC321349

[32] Newall F, Barnes C, Ignjatovic V, Monagle P (2003) Heparin-induced thrombocytopenia in children. J Paed Child Health 39: 289–292. 10.1046/j.1440-1754.2003.00139.x 12755937

[33] Weber C, Springer TA (1997) Neutrophil accumulation on activated, surface-adherent platelets in flow is mediated by interaction of Mac-1 with fibrinogen bound to alphaIIbbeta3 and stimulated by platelet-activating factor. J Clin Invest 100: 2085–2093. 9329974 10.1172/JCI119742 PMC508400

[34] Simon DI, Chen Z, Xu H, Li CQ, Dong J, et al (2000) Platelet glycoprotein ibalpha is a counterreceptor for the leukocyte integrin Mac-1 (CD11b/CD18). J Exp Med 192: 193–204. 10899906 10.1084/jem.192.2.193 PMC2193258

[35] Michelson AD, Barnard MR, Hechtman HB, MacGregor H, Connolly RJ, et al (1996) In vivo tracking of platelets: circulating degranulated platelets rapidly lose surface P-selectin but continue to circulate and function. Proc Natl Acad Sci U S A 93: 11877–11882. 8876231 10.1073/pnas.93.21.11877 PMC38152

[36] Hoffman RA (2005) Standardization, calibration, and control in flow cytometry. Curr Protoc Cytom Chapter 1: Unit 1 3. 10.1002/0471142956.cy0103s32 18770811

